# Involvement of mechano-sensitive Piezo1 channel in the differentiation of brown adipocytes

**DOI:** 10.1186/s12576-022-00837-1

**Published:** 2022-06-20

**Authors:** Manato Kenmochi, Satoko Kawarasaki, Satsuki Takizawa, Kazuhiko Okamura, Tsuyoshi Goto, Kunitoshi Uchida

**Affiliations:** 1 Graduate School of Integrated Pharmaceutical and Nutritional Sciences, University of Shizuoka, Shizuoka, 422-8526 Japan; 2 Division of Food Science and Biotechnology, Graduate School of Agriculture, Kyoto University, Uji, 611-0011 Japan; 3 Laboratory of Functional Physiology, Department of Environmental and Life Sciences, School of Food and Nutritional Sciences, University of Shizuoka, Yada 52-1, Suruga-ku, Shizuoka, 422-8526 Japan; 4 Department of Morphological Biology, Fukuoka Dental College, Fukuoka, 814-0193 Japan

**Keywords:** Brown adipocyte, Piezo channel, Differentiation, Calcineurin pathway

## Abstract

Brown adipocytes expend energy via heat production and are a potential target for the prevention of obesity and related metabolic disorders. Piezo1 is a Ca^2+^-permeable non-selective cation channel activated by mechanical stimuli. Piezo1 is reported to be involved in mechano-sensation in non-sensory tissues. However, the expression and roles of Piezo1 in brown adipocytes have not been well clarified. Here, we generated a brown adipocyte line derived from UCP1-mRFP1 transgenic mice and showed that Piezo1 is expressed in pre-adipocytes. Application of Yoda-1, a Piezo1 agonist, suppressed brown adipocyte differentiation, and this suppression was significantly attenuated by treatment with a Piezo1 antagonist and by Piezo1 knockdown. Furthermore, the suppression of brown adipocyte differentiation by Yoda-1 was abolished by co-treatment with a calcineurin inhibitor. Thus, these results suggest that activation of Piezo1 suppresses brown adipocyte differentiation via the calcineurin pathway.

**Supplementary Information:**

The online version contains supplementary material available at 10.1186/s12576-022-00837-1.

## Background

Adipose tissue, including white and brown adipose tissues (WAT and BAT, respectively), plays important roles in energy homeostasis [1]. White adipocytes contain single large lipid droplet that accumulate lipids for energy storage. On the other hand, brown adipocytes have multiple small lipid droplets and a high density of mitochondria, which convert energy to heat via proton transport across the inner mitochondrial membrane by uncoupling protein 1 (UCP1) [2], in response to sympathetic nervous system activation. This heat production and energy expenditure occur during cold exposure for maintenance of body temperature, known as non-shivering thermogenesis. BAT is distributed in the interscapular region and around the kidneys. Newborns have a high proportion of BAT, which gradually decreases with age [3, 4]. In 2009, using high-resolution imaging techniques, some studies demonstrated the existence of BAT not only in newborn, but also in adult humans [5–7]. Therefore, elucidation of the molecular mechanisms of BAT regulation and differentiation is thought to contribute to the treatment of obesity and obesity-related diseases in human.

Brown adipocytes originate from *Myf-5*-positive myoblasts, which differentiate into brown adipocytes upon expression of genes related to brown adipocyte functions, downstream of PRD1-BF1-RIZ1 homologous domain-containing protein 16 (PRDM16) expression [8]. Adipogenesis of both white and brown adipocytes requires peroxisome proliferator-activated receptor γ (PPARγ) and CCAAT/enhancer-binding protein α (C/EBPα) expression. Thus, differentiation of brown adipocytes results from increase in expression of not only thermogenesis-related genes, but also adipogenesis-related genes [9–11]. In both white and brown adipocytes, differentiation is negatively regulated by Ca^2+^ [12–15], and in white adipocytes, an increase in the intracellular Ca^2+^ concentration ([Ca^2+^]_i_) is thought to suppress differentiation via activation of calcineurin or calmodulin [16, 17]. However, the precise mechanisms underlying [Ca^2+^]_i_-dependent modulation of brown adipocyte differentiation are not fully understood.

Piezo channels were identified as mechano-sensitive cation channels by Patapoutian et al. in 2010 [18]. Piezo channels have a trimer structure shaped like a three-bladed propeller [19] and are expressed in a wide range of mechanically sensitive tissues [20]. Piezo1 is expressed mainly in non-sensory tissues, and it senses the shear stress in the endothelium of blood vessels and the storage pressure of urine in the bladder [21, 22]. Piezo2 is distributed predominantly in the cells of sensory tissues, such as dorsal root ganglion neurons and Merkel cells, and it contributes to touch sensation [23, 24]. Piezo1 is expressed in WAT, and adipocyte-specific knockout of Piezo1 in mice fed a high-fat diet resulted in increased WAT volume and a decreased number of white adipocytes, compared with wild-type mice fed a high-fat diet [25]. On the other hand, while Piezo1 is also expressed in BAT [26], the physiological roles of Piezo1 in brown adipocytes have not been well clarified. In addition, some reports have demonstrated that mechanical stimuli modulate white and brown adipocyte differentiation [27–29]. Accordingly, we hypothesized that Piezo1 is expressed in brown adipocytes and involved in brown adipocyte differentiation.

In this study, we generated a brown adipocyte line from UCP1-mRFP1 transgenic mice [30] and found the expression of Piezo1 in brown adipocytes. Furthermore, we observed the effect of a Piezo1 agonist on the differentiation of brown adipocytes and analyzed the effect of Piezo1 knockdown (KD) in brown adipocytes.

## Material and methods

### Animals

Male C57Bl/6NCr mice (SLC, Hamamatsu, Japan) and UCP1-mRFP1 transgenic mice [30] were housed in a controlled environment (12-h light/dark cycle; 22–24 °C; 50–60% humidity) with food and water provided ad libitum. All animal protocols were approved by the animal research committees of University of Shizuoka (Shizuoka, Japan) and Kyoto University (Kyoto, Japan), and were performed in accordance with institutional guidelines.

### Brown adipocyte line

We used immortalized pre-adipocytes isolated from interscapular BAT (iBAT) of UCP1-mRFP1 transgenic mice [30]. Immortalized pre-adipocytes were cultured in standard medium containing FBS, glutamine, and penicillin/streptomycin in DMEM. After reaching confluence, pre-adipocytes were incubated in standard medium supplemented with 10 μg/mL insulin, 1 nM triiodothyronine (T3), 0.125 mM indomethacin, 0.25 μM dexamethasone, and 0.5 mM 3-isobutyl 1-methylxanthine from days 0 to 2 for induction. Following induction, the induction medium was changed to differentiation medium consisting of standard medium supplemented with 5 μg/mL insulin and 1 nM T3, and the adipocytes were cultured for 6 additional days (from days 2 to 8). For the treatments, compounds were added to the cell medium from days 0 to 8 or from days 2 to 8; the same volume of solvent used to administer the compounds was added to the medium for the control treatment.

### Reverse-transcription PCR

Total RNA was purified from the brown adipocytes and iBAT of male C57Bl/6NCr mice using NucleoSpin RNA (Macherey–Nagel GmbH & Co., Duren, Germany) according to the manufacturer’s protocol. Reverse-transcription polymerase chain reaction (RT-PCR) was performed using the PrimeScript RT Reagent Kit (Takara Bio Inc., Shiga, Japan) and Taq DNA polymerase (New England Biolabs, Ipswich, MA, USA). The primer sequences are listed in Table 1.

**Table 1:** Sequences of the primers for RT-PCR and RT-qPCR

Gene	Forward primer (5′–3′)	Reverse primer (5′–3′)
*Piezo1* (RT-PCR)	CGGAACCTGACCTTGACAAC	CCAACTGGTGCAGGCTGAC
*Act-b* (RT-PCR)	ACCCGCGAGCACAGCTTCT	ATCACACCCTGGTGCCTA
*Ucp1* (RT-qPCR)	CAAAGTCCGCCTTCAGATCC	AGCCGGCTGAGATCTTGTTT
*Piezo1* (RT-qPCR)	ATCCTGCTGTATGGGCTGAC	AAGGGTAGCGTGTGTGTTCC
*Pparγ* (RT-qPCR)	GTGCCAGTTTCGATCCGTAGA	GGCCAGCATCGTGTAGATGA
*Prdm16* (RT-qPCR)	CAGCACGGTGAAGCCATTC	GCGTGCATCCGCTTGTG
*Pgc1α* (RT-qPCR)	CCCTGCCATTGTTAAGACC	TGCTGCTGTTCCTGTTTTC
*Caspase-3* (RT-qPCR)	GGAGCTTGGAACGGTACGC	CACATCCGTACCAGAGCGAG
*Bax* (RT-qPCR)	GAGCTGCAGAGGATGATTGC	CTTGGATCCAGACAAGCAGC
*Bcl-2* (RT-qPCR)	GTCGCTACCGTCGTGACTTC	CTGGGGCCATATAGTTCCACAA
*36b4* (RT-qPCR)	GGCCCTGCACTCTCGCTTTC	TGCCAGGACGCGCTTGT

### Quantitative reverse-transcription PCR

Gene copy numbers were determined by quantitative RT-PCR (RT-qPCR) using THUNDERBIRD SYBR qPCR Mix (Toyobo Co., Oosaka, Japan) following the manufacturer’s protocol. Data were collected during each extension phase of PCR and analyzed using the StepOne™ Real-Time PCR System (Thermo Fisher Scientific Inc., Waltham, MA, USA) or LightCycler480 System II (Roche Diagnostics, Mannheim, Germany). The results were standardized for comparison by measuring the mRNA level of *36b4* in each sample. The primer sequences are listed in Table 1.

### Immunohistochemical analysis of brown adipose tissue

BAT blocks from male C57Bl/6NCr mice fixed in 10% neutral buffered formalin and embedded in paraffin were cut into 3-μm-thick sections for hematoxylin and eosin (H&E) and immunohistochemical staining. Antigen retrieval was performed by autoclave treatment at 121 °C for 5 min in 0.01 M citrate buffer, pH 6.0. Immunostaining was performed using the EnVision/horseradish peroxidase kit (DAKO/Agilent Technologies Co., Santa Clara, CA, USA). Briefly, the sections were treated with 0.1% hydrogen peroxide/methanol solution to inhibit endogenous peroxidase activity and then with 5% goat normal serum in PBS to block any non-specific binding of primary antibodies. Subsequently, each section was incubated with a primary rabbit polyclonal antibody against UCP1 (1:250 dilution; #ab10983, Abcam, Cambridge, UK) or Piezo1 (1:200; #15,939-1-AP, Proteintech, Rosemont, IL, USA) at 20 °C overnight. After washing in PBS, the sections were incubated with a horseradish peroxidase-conjugated anti-rabbit secondary antibody. Peroxidase activity was visualized using 0.1% 3,3ʹ-diaminobenzidine and 0.01% hydrogen peroxide in PBS. Images were obtained using the ECLIPSE 50i microscope (Nikon Corporation, Tokyo, Japan) coupled with the imaging software (NIS elements; Nikon Corporation).

### Ca^2+^-imaging

[Ca^2+^]_i_ was monitored by loading each sample with Fluo-4 fluorescent dye (Thermo Fisher Scientific Inc.). Each sample was incubated with 5 M Fluo-4 AM for more than 30 min and used in experiments within 3 h. Fluorescent signals were collected using the CoolSNAP ES CCD camera (Photometrics, Tucson, AZ, USA) and recorded using NIS Elements software at 5-s intervals. The bath solution contained 140 mM NaCl, 5 mM KCl, 2 mM MgCl_2_, 2 mM CaCl_2_, 10 mM HEPES, and 10 mM glucose, pH 7.4, adjusted with NaOH. Cell viability was confirmed using 5 μM ionomycin. The fluorescence intensity was analyzed using NIS Elements software and Image J software (National Institutes of Health, Bethesda, MD, USA). The change in the fluorescence intensity value (ΔF_*Norm*_) was normalized using the following formula:

$$ \Delta {\text{F}}_{Norm} \left( \% \right) = \left( {{\text{F}} - {\text{F}}_{Initial} } \right)/\left( {{\text{F}}_{Inomycin} - {\text{F}}_{Initial} } \right) \times 100, $$

where F_*Initial*_ is the fluorescence intensity of each cell during the first 30 s of the experiment, and F_*Ionomycin*_ is the maximum fluorescence intensity during ionomycin application. In this study, ΔF_*Norm*_ ≥ 30% in cells treated with Yoda-1 (a Piezo1 agonist) was regarded as a positive response to Yoda-1. Cells that did not respond to ionomycin application were excluded from the analysis. All experiments were performed at room temperature.

### Oil red O staining and measurement of the triglyceride level

Oil red O staining was performed using Oil Red O dye (Nacalai Tesque, Inc., Kyoto, Japan). In brief, differentiated brown adipocytes on day 8 were fixed in 4% formalin and incubated at room temperature for more than 1 h. After fixation, the cells were washed twice with purified water and then with 60% isopropanol at room temperature for 5 min. The cells were dried completely and incubated with Oil red O solution at room temperature for 10 min. Oil red O solution was removed by addition of purified water, and the cells were washed four times with purified water. Images were acquired under a microscope (Keyence Corporation, Osaka, Japan). To measure triglyceride levels, all water was removed, and the cells were dried completely. Oil red O dye was eluted by incubation with 100% isopropanol at room temperature for 10 min. The OD values were measured at 490 nm using a microplate reader (Corona Electric Co., Hitachinaka, Japan); 100% isopropanol was used as the blank. The inhibition ratio of Yoda-1 treatment was calculated by dividing the absorbance of Yoda-1-treated cells by the absorbance of vehicle-treated cells.

### Knockdown of *Piezo1* by siRNA

siRNA was designed against mouse *Piezo1* (target sequence: TCGGCGCTTGCTAGAACTTCA) as reported previously [21]. Pre-adipocytes were transfected with 40 nM siRNA using Lipofectamine RNAiMAX (Thermo Fisher Scientific Inc.) for 24 h and then incubated with standard medium. After 48 h, the medium was changed to induction medium. To confirm KD efficiency, *Piezo1* mRNA expression was measured by RT-qPCR after 72 h of transfection.

### Total genomic DNA measurement

After pharmacological treatment, genomic DNA was extracted from differentiated brown adipocytes on day 8 using NucleoSpin DNA RapidLyse (Macherey–Nagel GmbH & Co.), following the manufacturer’s instructions. The OD values were measured at 260 nm using the multi-mode reader SYNERGY HTX (BioTec Instruments, Inc., Winooski, VT, USA).

### Propidium iodide staining

After pharmacological treatment, the cells were treated with trypsin–EDTA (Thermo Fisher Scientific Inc.), and the collected cells were washed with PBS. The cells (1 × 10^6^/100 μL) were incubated with propidium iodide (Nacalai Tesque, Inc.) for 15 min at room temperature. Then, the cells were plated on glass-bottom dishes, and images were obtained using a microscope (ECLIPSE Ti2; Nikon Corporation) coupled with NIS elements software.

### Calcineurin activity assay

A calcineurin activity assay was performed using the Calcineurin Cellular Activity Assay Kit (Enzo Life Sciences, Inc., Farmingdale, NY, USA), following the manufacturer’s instructions. DMSO, 10 μM Yoda-1, or 10 μM Yoda-1 with 20 μM Dooku-1 was applied to pre-adipocytes during induction. Cell lysates were collected and desalted from the induced adipocytes. Phosphopeptide substrate was applied to the cell lysates, and dephosphorylation was induced by incubating at 30 °C for 30 min. After reaction with BIOMOL Green reagent, the OD values were measured at 620 nm using a microplate reader (Corona Electric Co.). Calcineurin activity was calculated by the following equation:

$$ {\text{Calcineurin}}\,{\text{activity}}\,\left( {{\text{nmol}}_{{{\text{PO4}}}} } \right) = {\text{Total}}\,{\text{phosphate}}\,{\text{released }} - {\text{ Phosphate}}\,{\text{released}}\,{\text{under}}\,{\text{Ca}}^{{2 + }} {\text{-chelated}}\,{\text{conditions}}{.} $$

### Statistical analysis

All data are presented as means + SEM. Statistical analysis was performed using one-way ANOVA followed by multiple *t*-tests with Bonferroni correction, two-way ANOVA followed by Student’s *t*-test, or Student’s *t*-test using Origin 8.5 software (OriginLab Corporation, Northampton, MA, USA). p values less than 0.05 were considered to represent significant differences.

## Results

### UCP1-mRFP1 brown adipocytes have a high differential capacity

We isolated primary pre-adipocytes from the iBAT of a brown adipocyte transgenic mouse model, UCP1-mRFP1 (Additional file 1: Figure S1A). For immortalization, the pre-adipocytes were transformed with the SV40 large T antigen, and the resulting immortalized pre-adipocytes were cloned (Additional file 1: Figure S1A). Four clonal brown pre-adipocyte lines underwent differentiation, and their characteristics were evaluated. Oil Red O staining showed similar extents of lipid accumulation in all clones (Additional file 1: Figure S1B). All clones demonstrated the increases in *Ucp1* mRNA expression after treatment with 10 μM isoproterenol, a β-adrenergic receptor agonist, or 0.5 μM rosiglitazone, a PPARγ agonist (Additional file 1: Figure S1C). Since clone #1 exhibited the best response to both stimuli among the four clones, clone #1 was used in the experiments. In clone #1, the UCP1 protein levels were increased by rosiglitazone or isoproterenol treatment (Additional file 1: Figure S1D). On the other hand, although the mRFP1 protein level was increased by rosiglitazone and isoproterenol treatment (Additional file 1: Figure S1D), the mRFP1 fluorescent signal was not observed after treatment (Additional file 1: Figure S1E). Next, we confirmed the expression of genes related to the differentiation of brown adipocytes by RT-qPCR. The expression of *Pparγ* in inducted adipocytes (Day 2) and differentiated brown adipocytes on day 4 was significantly increased compared with that in pre-adipocytes (Additional file 1: Figure S2). The expression of *Pparγ* in differentiated brown adipocytes on day 8 tended to be higher than that in pre-adipocytes (Additional file 1: Figure S2). On the other hand, the expression of *Prdm16* and PPARγ coregulator 1α (*Pgc1α*) was significantly increased during the differentiation of brown adipocytes (Additional file 1: Figure S2). Therefore, this cell line might not be useful as a reporter brown pre-adipocyte line, but it represents brown pre-adipocytes with a high differentiation ability and responsiveness to UCP1 inductive stimuli.

### Piezo1 is expressed in brown adipocytes

**Fig. 1:**
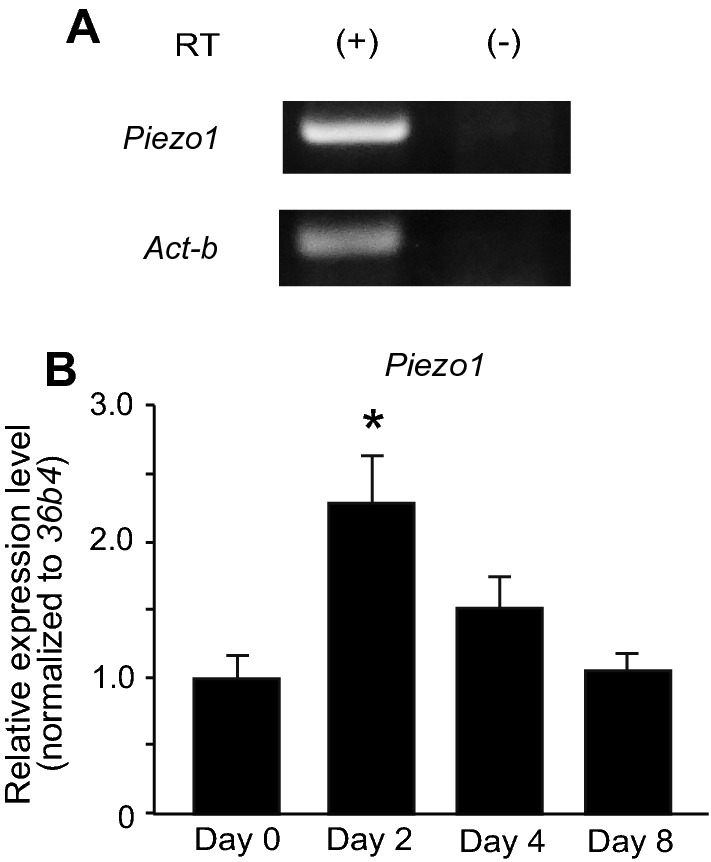
The mRNA expression of Piezo1 in mouse brown adipocytes. **A** RT-PCR analysis of *Piezo1* and *Act-b* expression in pre-adipocytes. RT ( +) and (−) indicate the samples treated with and without reverse transcriptase (RT), respectively. **B** RT-qPCR analysis of *Piezo1* expression in pre-adipocytes (Day 0), inducted adipocytes (Day 2), and differentiated brown adipocytes (Days 4 and 8). Each expression level was normalized to that of *36b4*. Each column represents the mean + SEM of 5 experiments. Statistical significance was assessed using ANOVA followed by two-tailed multiple *t*-tests with Bonferroni correction. *p < 0.05 vs. Day 0

First, we confirmed the mRNA expression of *Piezo1* in pre-adipocytes by RT-PCR (Fig. 1A). We next performed RT-qPCR to evaluate the change in *Piezo1* expression during the differentiation of brown adipocytes. As shown in Fig. 1B, *Piezo1* mRNA expression was temporarily increased in the inducted adipocytes (Day 2), followed by gradual decreases in the differentiated brown adipocytes on days 4 and 8.

**Fig. 2:**
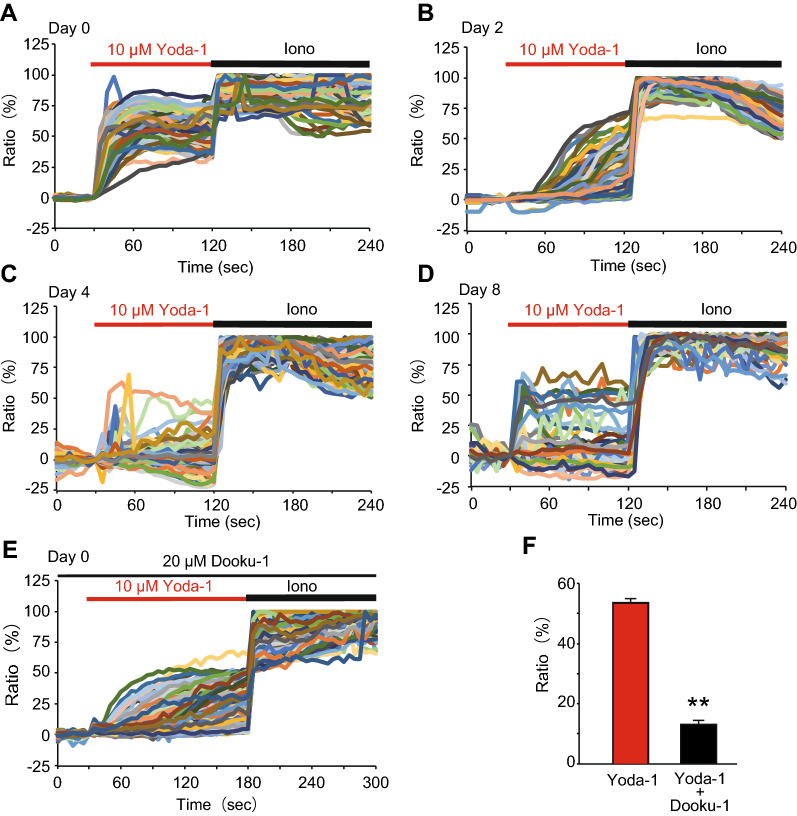
The functional expression of Piezo1 in brown adipocytes. **A–D** Representative Ca^2+^-imaging traces of changes in the intracellular Ca^2+^ concentration ([Ca^2+^]_i_) induced by 10 μM Yoda-1, a Piezo1 agonist, in pre-adipocytes (Day 0, **A**), inducted brown adipocytes (Day 2, **B**), and differentiated brown adipocytes on days 4 (**C**) and 8 (**D**). Ionomycin (5 μM, Iono) was used to confirm cell viability. **E** Representative traces of changes in [Ca^2+^]_i_ induced by 10 μM Yoda-1 with 20 μM Dooku-1, a Piezo1 antagonist, in pre-adipocytes (Day 0). **F** Summary of [Ca^2+^]_i_ changes 90 s after application of 10 μM Yoda-1 with or without 20 μM Dooku-1 in pre-adipocytes (Day 0). Each column represents the mean + SEM of 102–118 cells. Statistical significance was assessed using Student’s *t*-test. **p < 0.01 vs. Yoda-1

To examine the functional expression of Piezo1 in brown adipocytes, we performed Ca^2+^-imaging. An increase in [Ca^2+^]_i_ was observed after application of 10 μM Yoda-1, a Piezo1 agonist, in pre-adipocytes, and almost all cells (95.8%, 183/191 cells) responded to Yoda-1. Furthermore, the extent of the Yoda-1-induced [Ca^2+^]_i_ increase gradually decreased from day 0 (pre-adipocytes) to day 8 (differentiated brown adipocytes) (Fig. 2A–D). The population of Yoda-1-responding cells also decreased during differentiation (from 53.8% (283/526) in inducted adipocytes on day 2 to 4.9% (8/163) in differentiated brown adipocytes on day 4 and 11.5% (18/157) in differentiated brown adipocytes on day 8). To confirm the selectivity of Yoda-1, we observed the effect of a Piezo1 antagonist, Dooku-1, on the increased [Ca^2+^]_i_ induced by Yoda-1. Treatment with 20 μM Dooku-1 significantly reduced the [Ca^2+^]_i_ increase in pre-adipocytes (Day 0) treated with 10 μM Yoda-1 (Fig. 2E and F). These results suggest that Piezo1 is functionally expressed in pre-adipocytes, and its expression gradually decreases during differentiation.

### Piezo1 is expressed in mouse brown adipose tissue

**Fig. 3:**
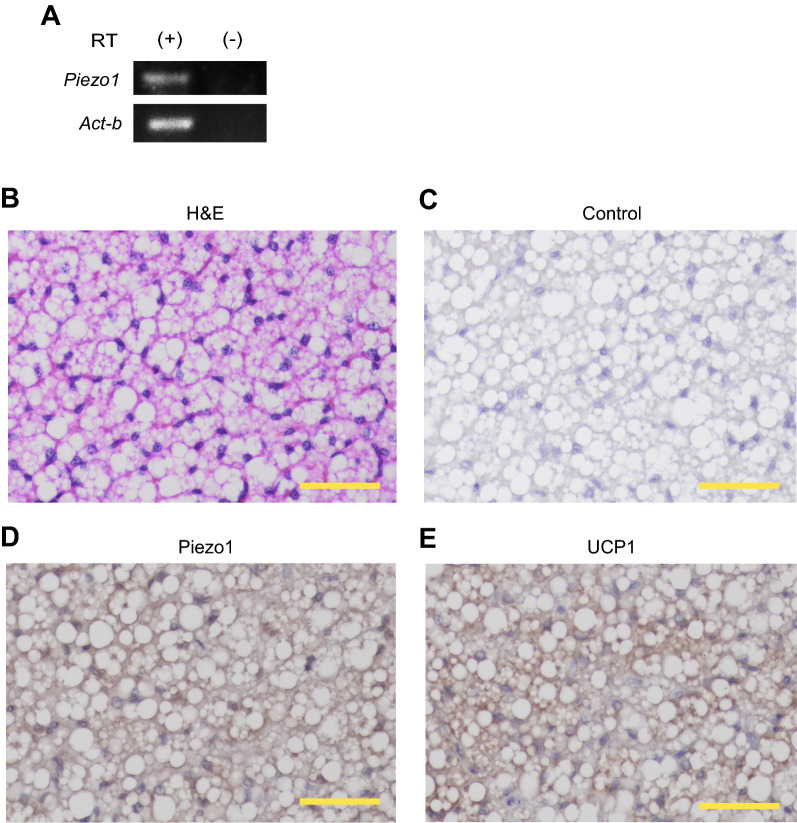
Piezo1 protein expression in mouse interscapular brown adipose tissue. **A** RT-PCR analysis of *Piezo1* and *Act-b* expression in mouse interscapular brown adipose tissue (iBAT) from 6-week-old male wild-type (WT) mice. RT ( +) and (−) indicate the samples treated with and without reverse transcriptase, respectively. **B** Morphological image of iBAT from 6-week-old male WT mice stained with hematoxylin and eosin (H&E). **C** Immunohistochemical image of iBAT from 6-week-old male WT mice in the absence of the primary antibody (Control). **D** and **E** Immunohistochemical images of iBAT from 6-week-old male WT mice using an anti-Piezo1 (**D**) or anti-UCP1 (**E**) antibody. Scale bar: 50 μm

We next assessed the expression of *Piezo1* in iBAT by RT-PCR, and confirmed the mRNA expression of *Piezo1* in iBAT (Fig. 3A). In addition, we evaluated the protein expression of Piezo1 by immunohistochemistry. First, we confirmed the presence of cells containing multiple small lipid droplets in iBAT by H&E staining (Fig. 3B). Next, we performed immunohistochemical staining using an anti-Piezo1 antibody. As shown in Fig. 3D, Piezo1 staining was observed around the lipid droplets. Furthermore, UCP1 staining was observed in the cytoplasm and around the lipid droplets (Fig. 3E). On the other hand, no staining was observed in the control lacking the primary antibody (Fig. 3C). These results suggest that Piezo1 is expressed in mouse iBAT.

### Activation of Piezo1 suppresses brown adipocyte differentiation

**Fig. 4:**
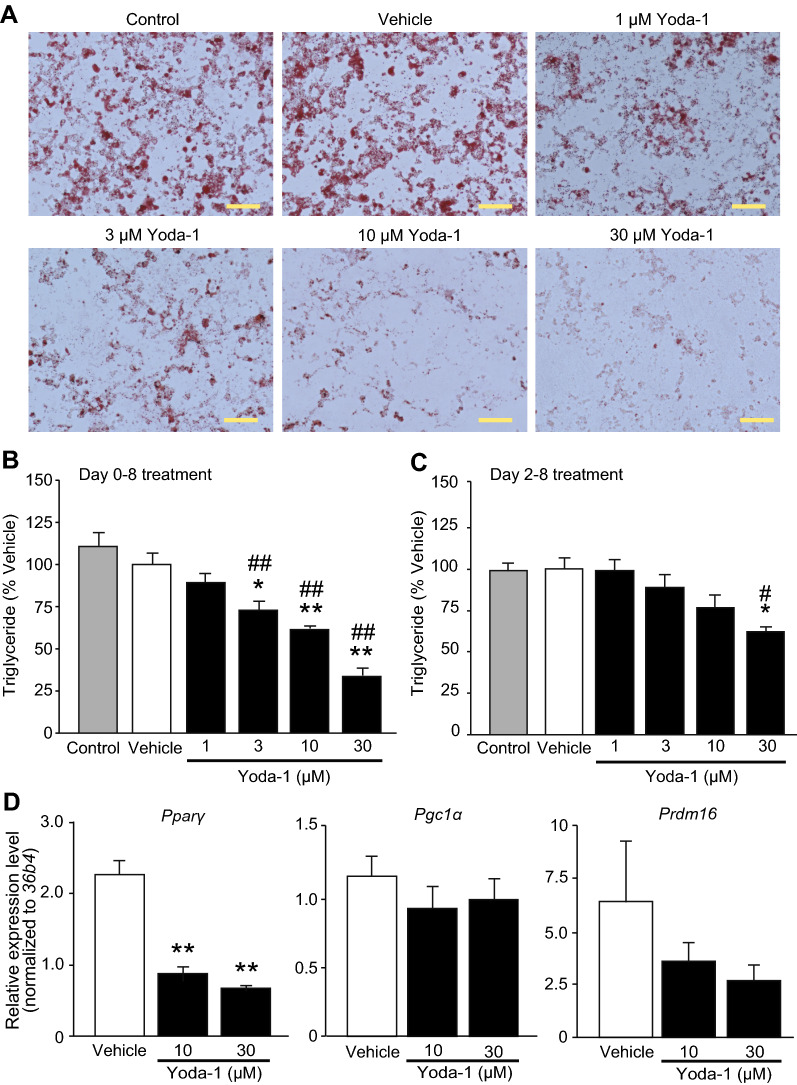
Application of a Piezo1 agonist suppresses the differentiation of brown adipocytes. **A** Representative images of Oil Red O staining of differentiated brown adipocytes treated with 1–30 μM Yoda-1 from days 0 to 8. Control denotes brown adipocytes in differentiation medium without the solvent. Vehicle denotes brown adipocytes in differentiation medium with solvent (0.1% DMSO). Scale bar: 100 μm. **B** The triglyceride levels in differentiated brown adipocytes treated with or without 1–30 μM Yoda-1 from days 0 to 8 (during induction and differentiation). The triglyceride levels were normalized to that in the vehicle treatment (absorbance; 0.280 ± 0.019). **C** The triglyceride levels in differentiated brown adipocytes treated with or without 1–30 μM Yoda-1 from days 2 to 8 (during differentiation). The triglyceride levels were normalized to that in the vehicle treatment (absorbance; 0.428 ± 0.029). **D** The changes in the mRNA levels of peroxisome proliferator-activated receptor γ (*Pparγ*, left), Pparγ coactivator1 α (*Pgc1α*, middle), and PRD1-BF1-RIZ1 homologous domain containing protein 16 (*Prdm16*, right) after 10 and 30 μM Yoda-1 treatment in differentiated brown adipocytes. Each expression level was normalized to that of *36b4*. Each column represents the mean + SEM of 5–7 experiments. Statistical significance was assessed using ANOVA followed by two-tailed multiple *t*-tests with Bonferroni correction. *p < 0.05, **p < 0.01 vs. Vehicle. ^#^*p* < 0.05, ^##^*p* < 0.01, vs. Control

**Fig. 5:**
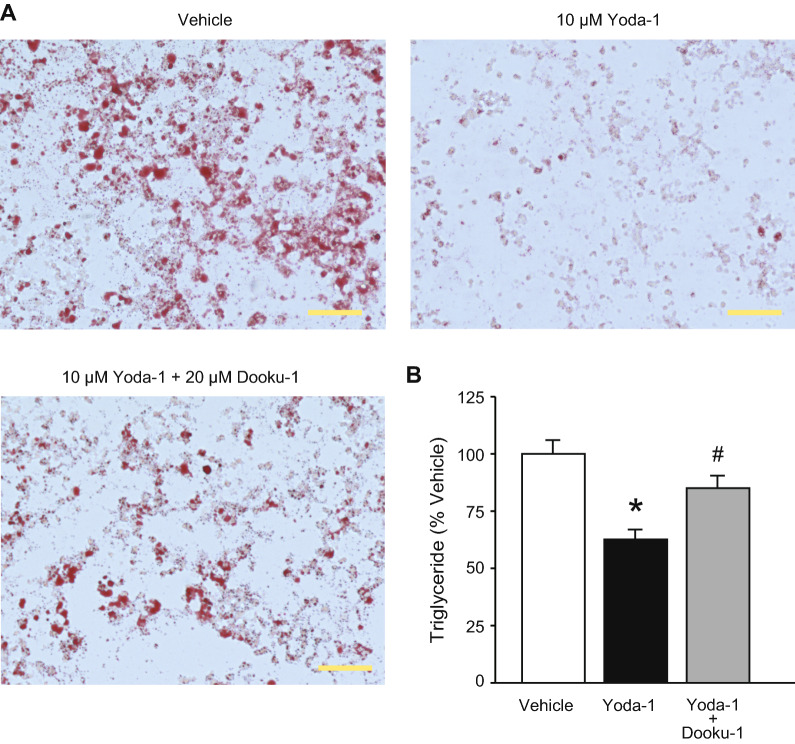
A Piezo1 antagonist prevents the suppression of brown adipocyte differentiation induced by a Piezo1 agonist. **A** Representative images of Oil Red O staining following application of 10 μM Yoda-1 with or without 20 μM Dooku-1 in differentiated brown adipocytes. Vehicle denotes brown adipocytes in differentiation medium with solvent (0.2% DMSO). Scale bar: 100 μm. **B** The triglyceride levels following application of 10 μM Yoda-1 with or without 20 μM Dooku-1 in differentiated brown adipocytes. The triglyceride levels were normalized to that in the vehicle (absorbance; 0.194 ± 0.012). Each column represents the mean + SEM of 8 experiments. Statistical significance was assessed using ANOVA followed by two-tailed multiple *t*-tests with Bonferroni correction. *p < 0.05, vs. Vehicle, ^#^*p* < 0.05 vs. Yoda-1

To clarify the involvement of Piezo1 in brown adipocyte differentiation, we performed a pharmacological study. We observed lipid accumulation by Oil red O staining to assess differentiation. While treatment with vehicle (solvent) did not affect Oil red O staining, treatment with 1–30 μM Yoda-1 from days 0 to 8 reduced Oil red O staining (Fig. 4A). The triglyceride level was significantly reduced by treatment with 3–30 μM Yoda-1 in a dose-dependent manner (Fig. 4B), consistent with the Oil red O staining results (Fig. 4A). Next, to examine whether suppression of differentiation via Piezo1 activation occurred during induction of brown adipocytes, we added Yoda-1 to the adipocytes during differentiation from days 2 to 8. Application of 1–10 μM Yoda-1 from days 2 to 8 did not affect the triglyceride levels, and only application of 30 μM Yoda-1 significantly reduced the triglyceride level (Fig. 4C). We then measured the expression levels of genes related to differentiation. As shown in Fig. 4D, the expression of *Pparγ*, but not *Pgc1α* or *Prdm16*, was significantly reduced in differentiated brown adipocytes treated with 10 or 30 μM Yoda-1 from days 0 to 8, compared with vehicle-treated differentiated brown adipocytes. To confirm cell viability after treatment with Yoda-1, we measured the total genomic DNA content and the expression of genes related to apoptosis. The total genomic DNA content did not differ among control adipocytes, vehicle-treated adipocytes, and 10 μM Yoda-1-treated adipocytes (Additional file 1: Figure S3A). The expression of *Caspase-3* and the expression ratio of Bcl-2-associated X protein (*Bax*) to B-cell lymphoma 2 (*Bcl-2*), all of which are increased in apoptotic cells, did not differ between vehicle-treated adipocytes and 10 μM Yoda-1-treated adipocytes (Additional file 1: Figure S3B and C). Moreover, the number of propidium iodide-positive dead cells also showed little difference among control adipocytes, vehicle-treated adipocytes, and 10 μM Yoda-1-treated adipocytes (Additional file 1: Figure S3D), suggesting that Yoda-1 does not affect cell viability. Next, we confirmed the selectivity of Yoda-1 using Dooku-1, a Piezo1 antagonist. Co-application of 20 μM Dooku-1 from days 0 to 8 led to partial recovery of the Oil red O staining intensity (Fig. 5A). Similarly, the triglyceride level reduced by 10 μM Yoda-1 was significantly prevented by co-application of 20 μM Dooku-1 (Fig. 5B). These results suggest that activation of Piezo1 suppresses the differentiation of brown adipocytes.

### Knockdown of *Piezo1* facilitates brown adipocyte differentiation and prevents the Yoda-1-induced suppression of brown adipocyte differentiation

**Fig. 6:**
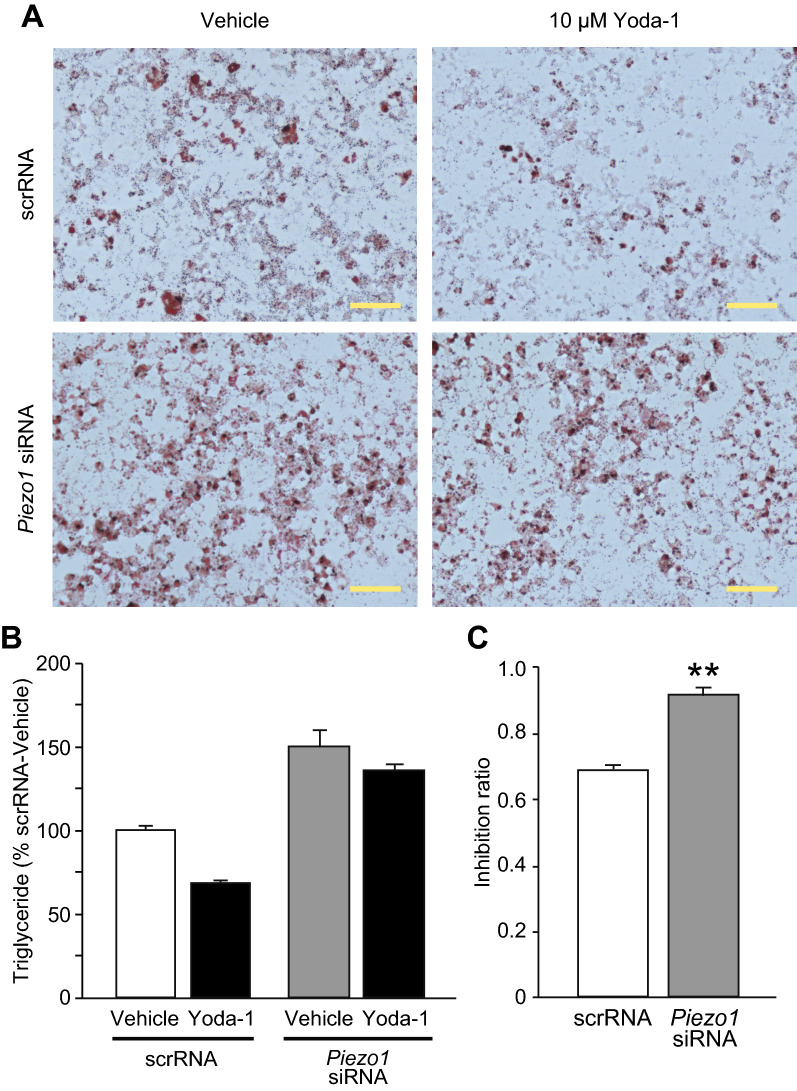
Knockdown of Piezo1 prevents the suppression of brown adipocyte differentiation by Yoda-1. **A** Representative images of Oil Red O staining in differentiated brown adipocytes transfected with scrRNA (upper) or *Piezo1* siRNA (lower). The cells were treated with (right) or without (Vehicle, left) 10 μM Yoda-1. Scale bar: 100 μm. **B** The triglyceride levels in differentiated brown adipocytes transfected with scrRNA or *Piezo1* siRNA, and treated with or without 10 μM Yoda-1. The triglyceride levels were normalized to that in the scrRNA-vehicle (absorbance; 0.219 ± 0.005). Each column represents the mean + SEM of 6 experiments. Statistical significance was assessed by two-way ANOVA (no significant interaction, *p* = 0.159), followed by Student’s *t*-test. scrRNA vs. *Piezo1* siRNA, *p* < 0.001; Vehicle vs. 10 μM Yoda-1, *p* = 0.0018. **C** The inhibition ratio calculated by dividing the absorbance of Yoda-1 by the absorbance of vehicle using the data in **B**. Each column represents the mean + SEM of 6 experiments. Statistical significance was assessed using Student’s *t*-test. **p < 0.01 vs. scrRNA

To further examine the involvement of Piezo1 in brown adipocyte differentiation, we analyzed *Piezo1*-KD adipocytes. First, we confirmed that *Piezo1* mRNA expression was drastically reduced in pre-adipocytes transfected with *Piezo1* siRNA (94.8%, n = 3). Using these *Piezo1*-KD adipocytes, we evaluated the effect of Yoda-1 on differentiation. As shown in Fig. 6A, although treatment with 10 μM Yoda-1 reduced Oil red O staining intensity in scrRNA-transfected differentiated brown adipocytes, this effect tended to be prevented in *Piezo1*-KD differentiated brown adipocytes. Furthermore, Oil red O staining appeared to be stronger in vehicle-treated *Piezo1*-KD adipocytes than in vehicle-treated scrRNA-transfected adipocytes (Fig. 6A). *Piezo1* KD also enhanced the triglyceride level in differentiated brown adipocytes (Fig. 6B). The reduction in the triglyceride level induced by 10 μM Yoda-1 was observed in both scrRNA-transfected and *Piezo1*-KD differentiated brown adipocytes (Fig. 6B). We then calculated the inhibition ratio of Yoda-1 treatment to rule out the possibility that the triglyceride level was enhanced by *Piezo1* KD. The inhibition ratio was significantly increased in *Piezo1*-KD adipocytes compared with scrRNA-transfected adipocytes (Fig. 6C), suggesting that suppression of adipocyte differentiation by Yoda-1 is reduced in *Piezo1*-KD adipocytes. These results strongly suggest that Piezo1 is involved in the differentiation of brown adipocytes.

### The calcineurin pathway potentially mediates the Yoda-1-induced suppression of brown adipocyte differentiation

**Fig. 7:**
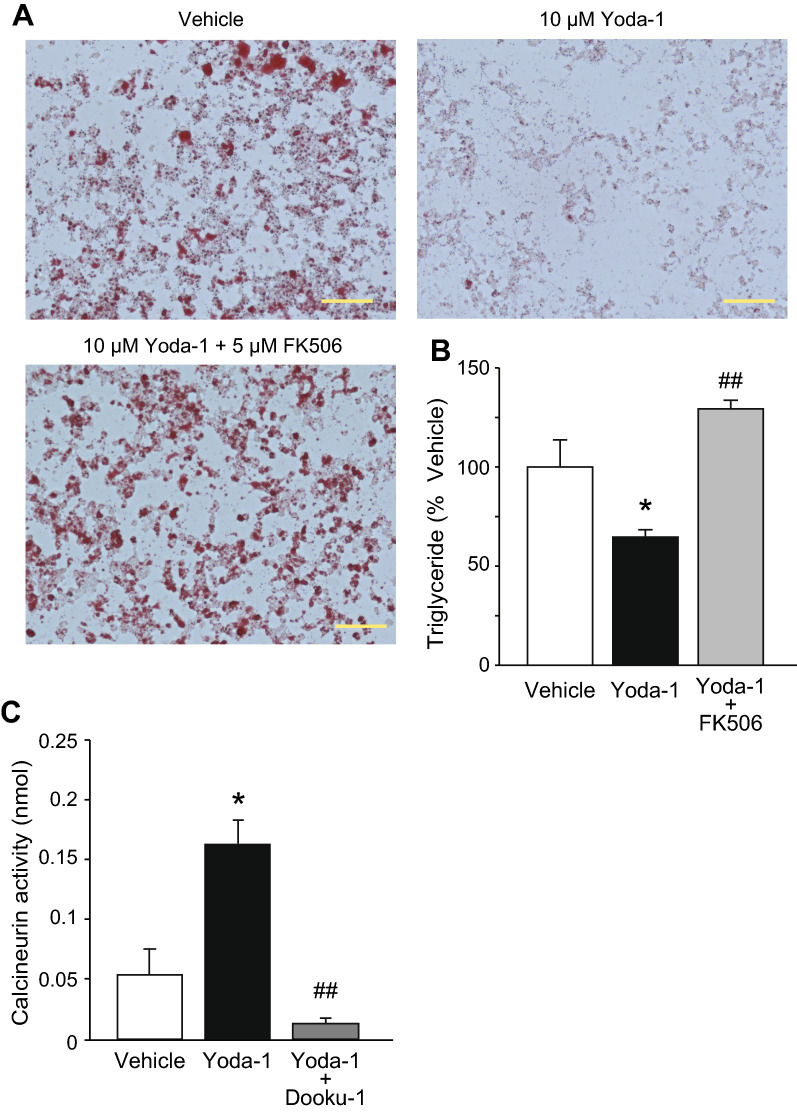
Treatment with a calcineurin inhibitor abolishes the inhibition of brown adipocyte differentiation by Yoda-1. **A** Representative images of Oil Red O staining in differentiated brown adipocytes following application of 10 μM Yoda-1 with or without 5 μM FK506. Vehicle indicates differentiation medium with solvent (0.2% DMSO). Scale bar: 100 μm. **B** The triglyceride levels following application of 10 μM Yoda-1 with or without 5 μM FK506 in differentiated brown adipocytes. Each data was normalized to that of vehicle (absorbance; 0.180 ± 0.024). Each column represents the mean + SEM of 5–7 experiments. Statistical significance was assessed using ANOVA followed by two-tailed multiple *t*-tests with Bonferroni correction. *p < 0.05 vs. vehicle, ^##^*p* < 0.01 vs. Yoda-1. **C** The calcineurin activities following application of 10 μM Yoda-1 with or without 20 μM Dooku-1 in inducted brown adipocytes. Each column represents the mean + SEM of 6–7 experiments. Statistical significance was assessed using ANOVA followed by two-tailed multiple *t*-tests with Bonferroni correction. *p < 0.05 vs. vehicle, ^##^*p* < 0.01 vs. Yoda-1

A previous study indicated that increases in [Ca^2+^]_i_ suppressed adipocyte differentiation via the calcineurin pathway [16]; therefore, we examined the effect of FK506, a calcineurin inhibitor, on the adipocyte differentiation suppressed by Yoda-1. Application of 5 μM FK506 abolished the reductions in the Oil red O staining intensity and triglyceride level induced by 10 μM Yoda-1 in differentiated brown adipocytes (Fig. 7A and B). Next, we examined whether the activation of Piezo1 enhances calcineurin activity in brown adipocytes. As shown in Fig. 7C, application of 10 μM Yoda-1 from days 0 to 2 increased the activation of calcineurin, and this increase was almost abolished by co-treatment with 20 μM Dooku-1. These results suggest that the calcineurin pathway is involved in Piezo1-mediated suppression of brown adipocyte differentiation.

## Discussion

In this study, we established a UCP1-mRFP1 transgenic brown adipocyte line, which has the ability to differentiate, from the BAT of UCP1-mRFP1 transgenic mice. We revealed that Piezo1 was expressed in these brown adipocytes, and that activation of Piezo1 suppressed brown adipocyte differentiation. Application of a Piezo1 antagonist and calcineurin inhibitor prevented Piezo1 agonist-mediated suppression of brown adipocyte differentiation. Furthermore, *Piezo1* KD facilitated the differentiation of brown adipocytes and prevented the Piezo1 agonist-induced suppression of their differentiation.

### The role of Piezo1 in brown adipocyte differentiation

Pharmacological activation of Piezo1 suppressed adipocyte differentiation (Fig. 4A and B). In white adipocytes, many reports have demonstrated that Ca^2+^ plays important roles in processes such as lipolysis and differentiation [13, 31, 32], and some Ca^2+^-permeable ion channels, including Piezo1, STIM1, and certain TRP channels, are expressed [26, 33, 34]. In brown adipocytes, there are a few reports on the roles of Ca^2+^ signaling and the expression of Ca^2+^-permeable ion channels [35]. Recently, Sun et al. demonstrated that an increase in [Ca^2+^]_i_ might suppress the differentiation of brown adipocytes, as evidenced by inhibition of brown adipocyte differentiation after application of thapsigargin or ionomycin, a calcium ionophore [29]. Therefore, in brown adipocytes, we believe that the increases in [Ca^2+^]_i_ induced by Piezo1 activation might suppress the differentiation of brown adipocytes. This notion could be supported by our findings that Dooku-1, an antagonist of Piezo1 activation induced by Yoda-1, prevented Yoda-1-induced suppression of differentiation (Fig. 5), and that *Piezo1* KD prevented Yoda-1-induced suppression of brown adipocyte differentiation (Fig. 6). We found that Piezo1 was functionally expressed in pre-adipocytes, and that its expression was reduced during differentiation (Fig. 2). Suppression of differentiation was weakened by application of Yoda-1 from days 2 to 8 compared with that from days 0 to 8 (Fig. 4B and C). In white adipocytes, increases in [Ca^2+^]_i_ induced by treatment with thapsigargin and a calcium ionophore during the early stage of white adipocyte differentiation inhibits differentiation [14, 15], indicating that increases in [Ca^2+^]_i_ during the early stage of white adipocyte differentiation suppresses differentiation. Although the significance of increased [Ca^2+^]_i_ during the early stage of brown adipocyte differentiation has not been well clarified, activation of Piezo1 during early differentiation could be important for the regulation of differentiation in brown adipocytes. On the other hand, *Piezo1* mRNA expression was temporally increased in brown adipocytes after induction (Fig. 1B), which is not consistent with the Ca^2+^-imaging data (Fig. 2A–D). Although it is difficult to explain the reasons for this discrepancy, some possibilities are that *Piezo1* mRNA is not translated into protein, or that Piezo1 protein is not transported to the plasma membrane. We suggest that the high expression of Piezo1 in the induction and early stages of differentiation might be involved in the suppression of brown adipocyte differentiation.

### Mechanisms of Piezo1-mediated suppression of brown adipocyte differentiation

The mRNA expression of *Pparγ*, but not *Pgc1α* or *Prdm16*, was significantly reduced by treatment with the Piezo1 agonist (Fig. 4D). *Prdm16* and *Pgc1α* mRNA expression was slightly increased upon differentiation, compared with *Pparγ* mRNA expression (Additional file 1: Figure S2). These genes are used as differentiation markers: *Pparγ* is a marker of adipogenesis in both white and brown adipocytes, and *Pgc1α* and *Prdm16* are markers of brown adipocyte differentiation [11]. Interestingly, activation of the calcineurin pathway suppresses differentiation and *Pparγ* and *C/ebpα* expression in white adipocytes (3T3-L1 cells) [16]. It is reported that *Pparγ* is expressed in early stage of differentiation in adipocyte [36, 37]. In addition, PPARγ activation during early-stage differentiation enhanced the differentiation of 3T3-L1 cells into white adipocytes [38, 39] and PPARγ activation during early-stage differentiation into white adipocytes was also enhanced in an embryonic fibroblast model of type 2 diabetes [40]. Those reports and our results suggest that Piezo1 suppresses differentiation by inhibiting *Pparγ* expression via the calcineurin pathway during the induction and early stage of differentiation. As another candidate for Ca^2+^-dependent cell signaling, Ca^2+^/calmodulin-dependent protein kinase (CaMKK2) is also reported to modulate adipogenesis via Ca^2+^ influx in white adipocytes [17]. As our study indicated that treatment with a calcineurin inhibitor completely prevented the suppression of differentiation (Fig. 7A and B) and that Piezo1 activation enhanced the calcineurin activity in inducted adipocytes (Fig. 7C), the calcineurin pathway may be mainly responsible for downstream of Piezo1 activation.

### The role of mechano-sensation in brown adipocyte function

It has been reported that mechanical stimuli (e.g., shear stress, stretch, and shaking) suppress the differentiation of both white and brown adipocytes [27–29]. Especially, stretching of 3T3-L1 cells during induction inhibited their differentiation by reducing *Pparγ* expression [41]. Taking into consideration our finding that Piezo1 activation suppresses adipocyte differentiation (Fig. 4), it is likely that mechanical stimuli can activate Piezo1, leading to impaired brown adipocyte differentiation. Our finding that KD of *Piezo1* enhanced lipid accumulation in brown adipocytes (Fig. 6) might suggest that endogenous membrane stretching, such as cell movement and migration, and the existence of endogenous modulators of Piezo1 activity (e.g., STOML3 [42] and/or unknown modulators in culture conditions) are sufficient to activate Piezo1 and modulate the differentiation of brown adipocytes. Under physiological conditions, it is also possible that similar mechanical stimuli could modulate brown adipocyte differentiation in BAT. The physiological significance of mechano-sensation via Piezo1 in BAT could be that it contributes to maintaining the number of differentiated brown adipocytes by sensing cell confluency and preventing lipid accumulation. However, further analyses including in vivo analyses are necessary to support this conclusion. It was also reported that a mechano-sensitive cation channel, TRPV2, is involved not only in brown adipocyte differentiation during early-stage differentiation, but also in non-shivering thermogenesis in differentiated brown adipocytes [29, 43]. Those reports and our results might indicate that brown adipocytes have multiple mechano-sensitive ion channels that modulate brown adipocyte function according to differentiation stage.

**Fig. 8:**
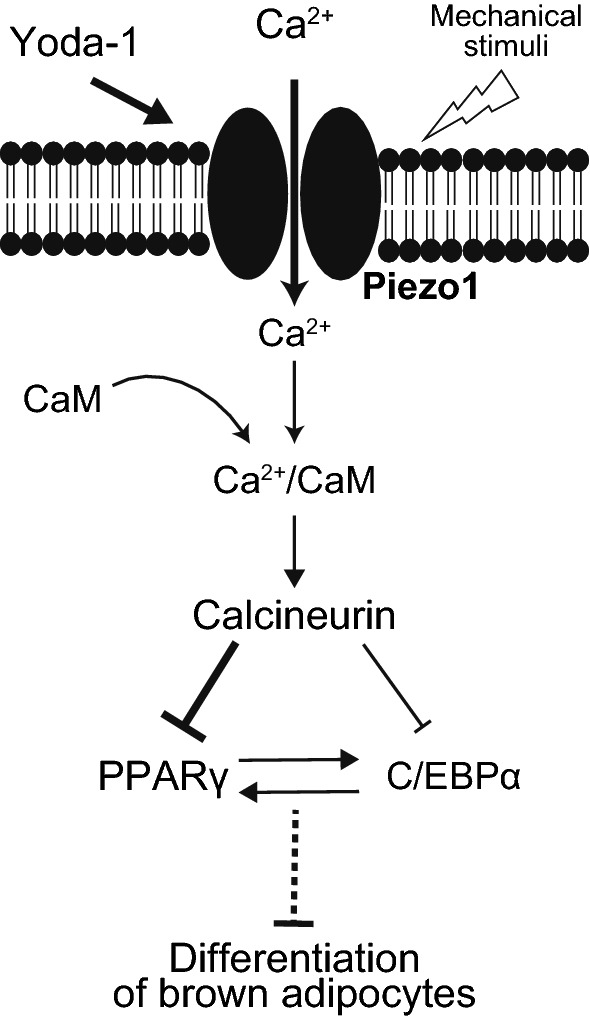
A proposed working model showing Piezo1-mediated suppression of differentiation in brown adipocytes. A Piezo1 agonist (Yoda-1) and endogenous stimuli including mechanical stimuli activate Piezo1, followed by an increase in the [Ca^2+^]_i_ in pre-adipocytes and the early stage of differentiation in brown adipocytes. Ca^2+^ binds to calmodulin (CaM), and the Ca^2+^/CaM complex activates calcineurin, which suppress the expression of the key transcriptional factors PPARγ and C/EBPα, which in turn suppress the differentiation of brown adipocytes

In conclusion, our study exhibited a novel role of Piezo1 in mouse brown adipocyte differentiation. Activation of Piezo1 during the induction and early stage of differentiation suppresses brown adipocyte differentiation via the calcineurin pathway (Fig. 8). Mechanical stimuli including cell stretching and migration and/or unknown modulators could lead to Piezo1 activation followed by suppression of differentiation. Thus, we propose that regulation of Piezo1 function could be a promising therapeutic approach for preventing and combating obesity and related metabolic disorders.

### Supplementary Information

**Additional file 1: Figure S1.** Establishment of UCP1-mRFP1 transgenic brown adipocytes and confirmation of their differentiation ability. (**A**) UCP1-mRFP1 transgenic brown adipocytes were established from the interscapular brown adipose tissue (iBAT) of UCP1-mRFP1 transgenic mice. (**B**) Confirmation of adipocyte differentiation in four established clones (#1 to #4) by Oil Red O staining. Scale bar: 200 μm. (**C**) *Ucp1* expression according to RT-qPCR in the established clones (#1 to #4). The four clones were treated with or without 10 μM isoproterenol (Iso, a β-adrenergic receptor agonist) and/or 0.5 μM rosiglitazone (Rosi, a PPARγ agonist). (**D**) Confirmation of the expression of UCP1 and mRFP1 proteins by Western blot analysis in UCP1-mRFP1 transgenic brown adipocytes (clone #1). β-actin was used as a positive control. (**E**) Confirmation of mRFP1 fluorescence in UCP1-mRFP1 transgenic brown adipocytes (clone #1). Scale bar: 200 μm. **Figure S2.** The changes in the gene expression in adipocytes during differentiation. RT-qPCR analysis of genes related to brown adipocyte differentiation in pre-adipocytes (Day 0), inducted adipocytes (Day 2), and differentiated brown adipocytes on days 4 and 8. Gene expression levels were normalized to those of *36b4*. Each column represents the mean + SEM of 5 experiments. Statistical significance was assessed using ANOVA followed by two-tailed multiple *t*-tests with Bonferroni correction. *p < 0.05, **p < 0.01 vs. Day 0. **Figure S3.** Confirmation of the viability of Yoda-1-treated brown adipocytes. (**A**) Total genomic DNA from differentiated brown adipocytes treated with or without 10 μM Yoda-1. Control represents brown adipocytes in differentiation medium without solvent. Vehicle represents brown adipocytes in differentiation medium with solvent (0.2% DMSO). (**B**) RT-qPCR analysis of *Caspase-3* expression in differentiated brown adipocytes treated with or without 10 μM Yoda-1. Gene expression levels were normalized to those of *36b4*. (**C**) RT-qPCR analysis of Bcl-2-associated X protein (*Bax*) expression relative to B-cell lymphoma 2 (*Bcl-2*) expression in differentiated brown adipocytes treated with or without 10 μM Yoda-1. Each column represents the mean + SEM of 5-6 experiments. (**D**) Images of propidium iodide (PI) staining of differentiated brown adipocytes treated with or without 10 μM Yoda-1 from days 0 to 8. Control represents brown adipocytes in differentiation medium without solvent. Vehicle represents brown adipocytes in differentiation medium with solvent (0.1% DMSO). PI staining was positive in 17.5% (76/434) of control brown adipocytes, 20.3% (110/543) of solvent-treated brown adipocytes, and 14.3% (96/672) of Yoda-1-treated brown adipocytes. BF: bright field. Scale bar: 100 μm.
