# Supplementary figures and images for: Involvement of mechano-sensitive Piezo1 channel in the differentiation of brown adipocytes
Source: J Physiol Sci. 2022 Jun 20;72:13. doi: 10.1186/s12576-022-00837-1 (PMC10717802; doi:10.1186/s12576-022-00837-1)

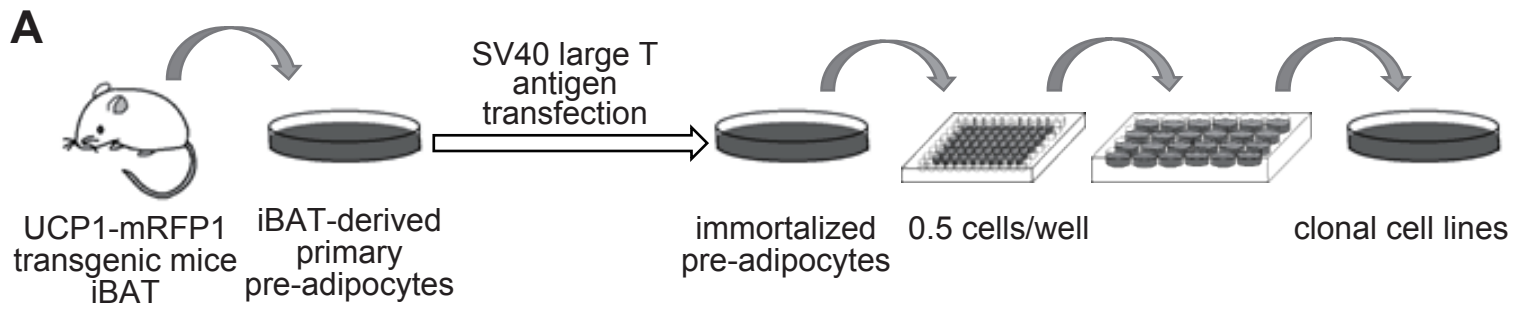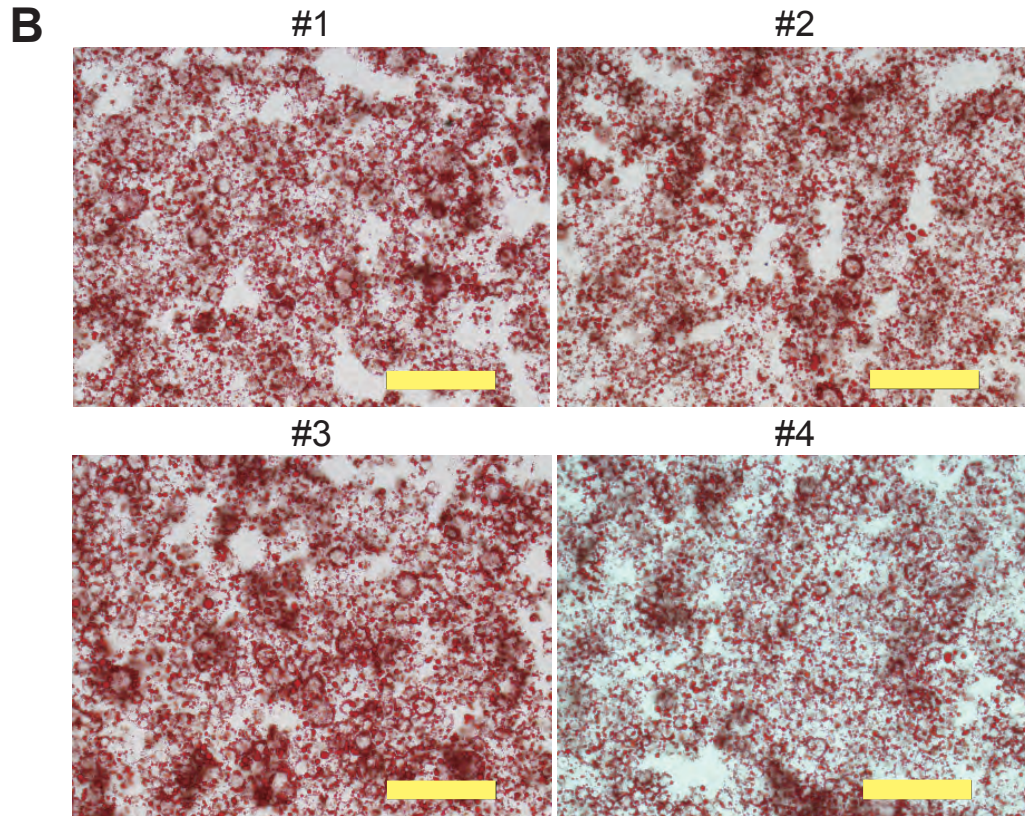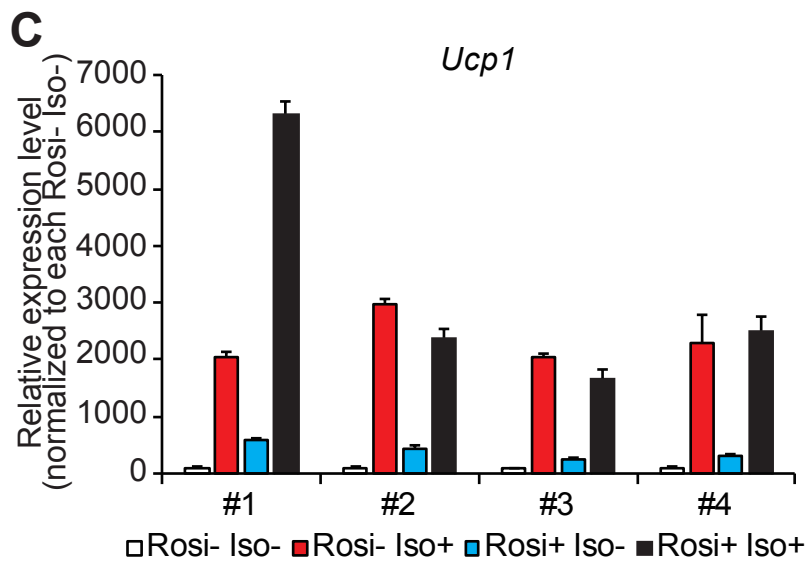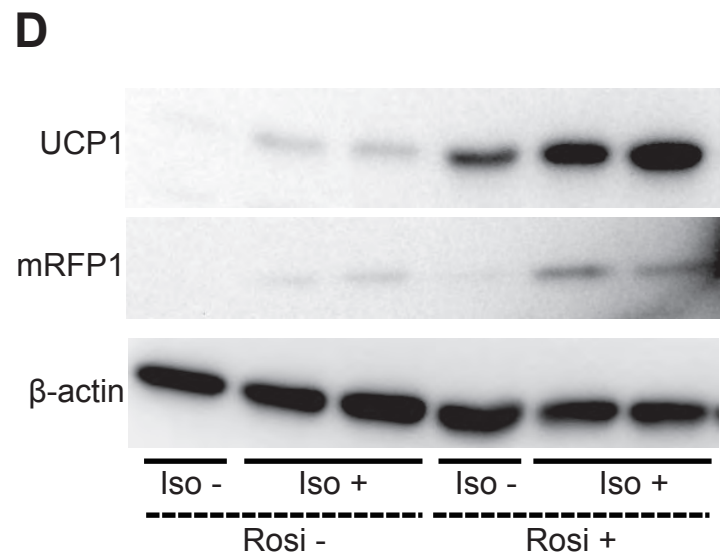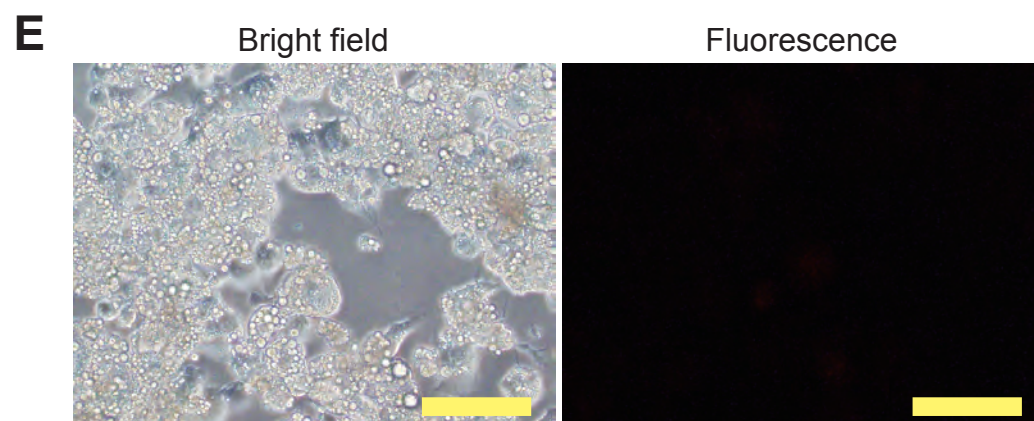

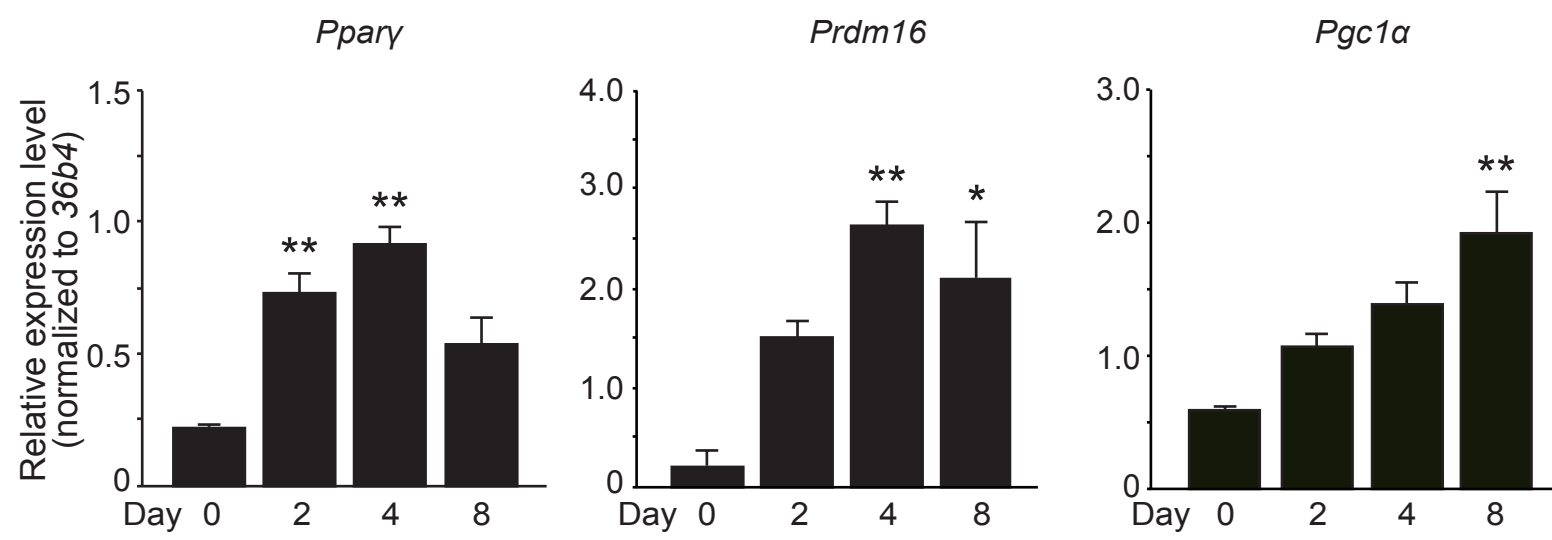

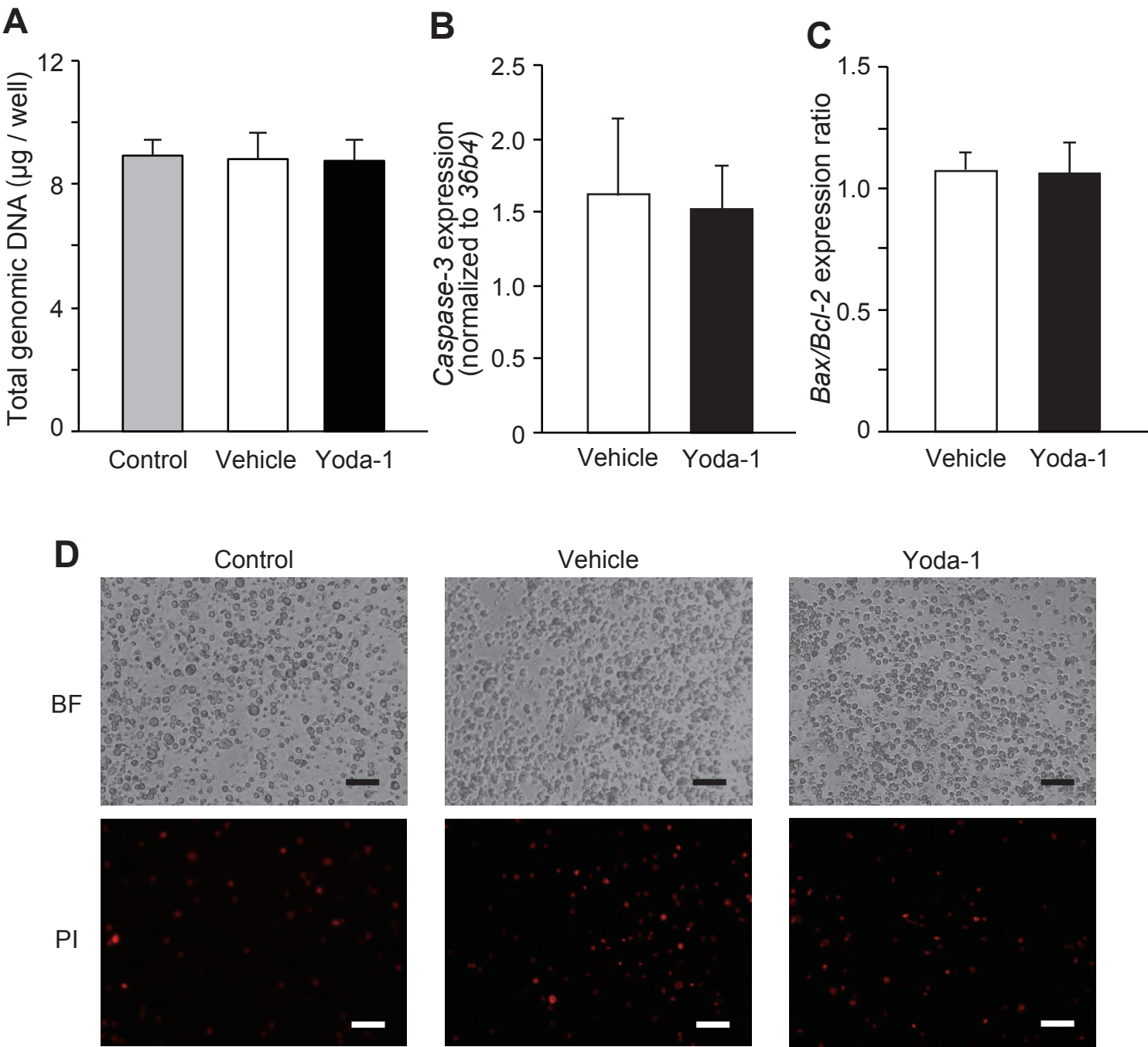

Supplement: Supplementary file 1 — Additional file 1: Figure S1. Establishment of UCP1-mRFP1 transgenic brown adipocytes and confirmation of their differentiation ability. (A) UCP1-mRFP1 transgenic brown adipocytes were established from the interscapular brown adipose tissue (iBAT) of UCP1-mRFP1 transgenic mice. (B) Confirmation of adipocyte differentiation in four established clones (#1 to #4) by Oil Red O staining. Scale bar: 200 μm. (C) Ucp1 expression according to RT-qPCR in the established clones (#1 to #4). The four clones were treated with or without 10 μM isoproterenol (Iso, a β-adrenergic receptor agonist) and/or 0.5 μM rosiglitazone (Rosi, a PPARγ agonist). (D) Confirmation of the expression of UCP1 and mRFP1 proteins by Western blot analysis in UCP1-mRFP1 transgenic brown adipocytes (clone #1). β-actin was used as a positive control. (E) Confirmation of mRFP1 fluorescence in UCP1-mRFP1 transgenic brown adipocytes (clone #1). Scale bar: 200 μm. Figure S2. The changes in the gene expression in adipocytes during differentiation. RT-qPCR analysis of genes related to brown adipocyte differentiation in pre-adipocytes (Day 0), inducted adipocytes (Day 2), and differentiated brown adipocytes on days 4 and 8. Gene expression levels were normalized to those of 36b4. Each column represents the mean + SEM of 5 experiments. Statistical significance was assessed using ANOVA followed by two-tailed multiple t-tests with Bonferroni correction. *p < 0.05, **p < 0.01 vs. Day 0. Figure S3. Confirmation of the viability of Yoda-1-treated brown adipocytes. (A) Total genomic DNA from differentiated brown adipocytes treated with or without 10 μM Yoda-1. Control represents brown adipocytes in differentiation medium without solvent. Vehicle represents brown adipocytes in differentiation medium with solvent (0.2% DMSO). (B) RT-qPCR analysis of Caspase-3 expression in differentiated brown adipocytes treated with or without 10 μM Yoda-1. Gene expression levels were normalized to those of 36b4. (C) RT-qPCR analysis of [file 12576_2022_837_MOESM1_ESM.pdf]
